# After hours admission to icu - impact on mortality

**DOI:** 10.1186/2197-425X-3-S1-A157

**Published:** 2015-10-01

**Authors:** HP Chan, Z Puthucheary, M Cove, A Mukhopadhyay, J Phua, HS Yip

**Affiliations:** National University Health System, Division of Respiratory and Critical Care Medicine, University Medicine Cluster, Singapore, Singapore; National University of Singapore, Department of Medicine, Yong Loo Lin School of Medicine, Singapore, Singapore

## Introduction

Patients admitted to intensive care units (ICU) after hours have higher mortality rates in several studies. This effect is, however, negated in most studies upon correction for disease severity.

## Objectives

We aim to explore differences in mortality between patients admitted during working hours (8am-5pm on weekdays and 8am-11am on weekends) compared to those admitted after hours.

## Methods

This is a retrospective cohort study of admissions to a medical ICU in a tertiary teaching hospital between 2010-2014. All patients with known APACHE II scores were analysed. Primary outcome measure was that of ICU mortality adjusted for disease severity and secondary outcome measure was length of ICU stay.

## Results

**Figure 1:**
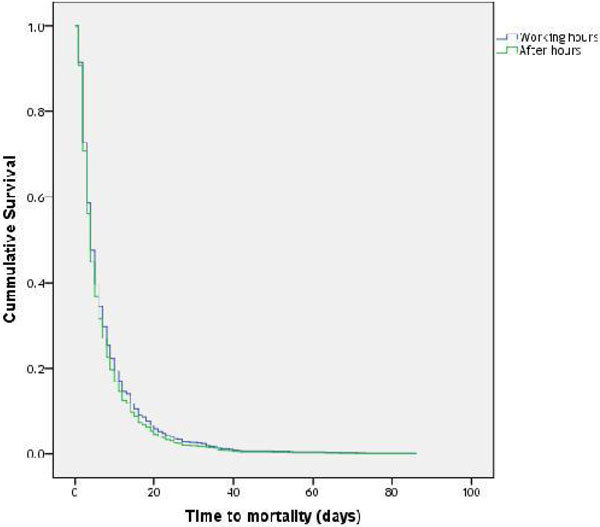
Figure 1

**Figure 2:**
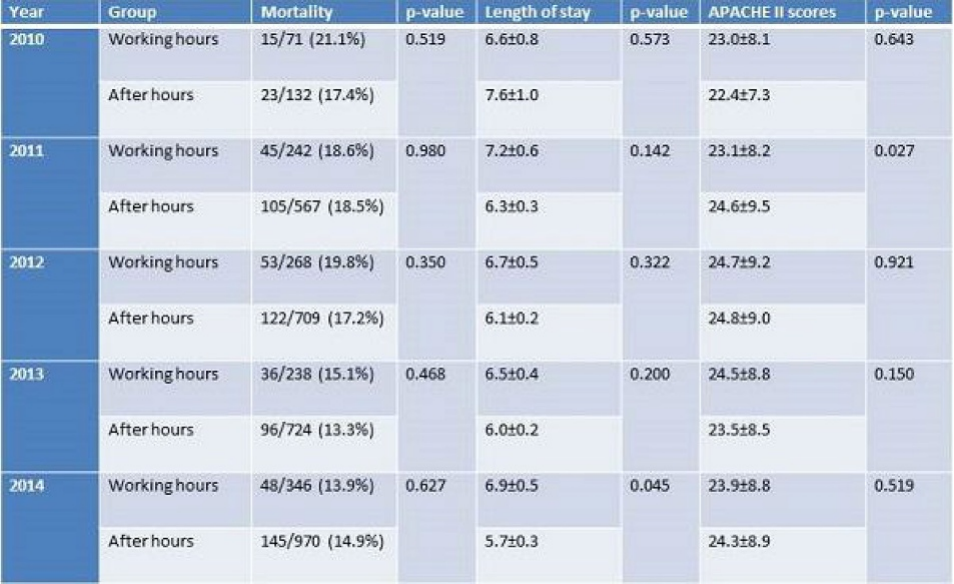
Figure 2

**Figure 3:**
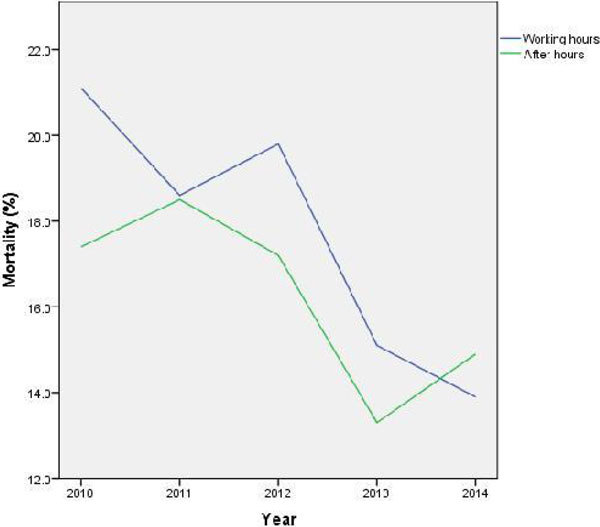
Figure 3

A total of 4266 admissions were included in this study. 1165 (27.3%) were admitted during working hours while 3101 (72.7%) were admitted after hours. There was no significant difference in APACHE II scores (24.0 ± 8.7 - working hours; 24.2 ± 8.9 - after hours, p = 0.492). Adjusted survival analysis did not demonstrate significant difference in mortality between the 2 groups (HR 0.923, 95%CI 0.782-1.089, p = 0.343) (figure 1). Patients admitted after hours have a significantly shorter ICU stay (5.8 ± 0.1 versus 6.5 ± 0.2, p = 0.007) if they survive their ICU stay. Year by year analysis also demonstrated no difference in mortality between cohorts (Figure 2) with a decrease in mortality seen in both in and out of hours admissions (figure 3).

## Conclusions

Timing of acute admission had no impact on ICU mortality over a 4 year period. These data which are contradictory to some published observational studies may reflect working patterns among our medical and nursing staff, and that traditional definitions of “in-hours” may need to be revisited. The lack of mortality difference provides reassurance that our current workflows, and acute management protocols are unaffected by working hours, and has ramifications for manpower planning.

